# Supplementary material for: Affection of Respiratory Muscles in ALS and SMA
Source: J Clin Med. 2022 Feb 22;11(5):1163. doi: 10.3390/jcm11051163 (PMC8910994; doi:10.3390/jcm11051163)
Supplement: Supplementary file 1 [file jcm-11-01163-s001.zip › Table S1.pdf]

Suppl. Table S1. Differences of diaphragm ultrasound imaging between the ALS and SMA subcohorts.

Parameter	Outcome	ALS (n=40)	SMA (n=23)	*P*-value§	Multiple logistic regression analysis#	
					Adjusted correlation coefficient (95%CI)	*P*-value
**Diaphragm thickness insp. right**	Path. (<2mm) [n/%]	7/40 (18%)	2/23 (9%)	.467	.647 (0.040-10.362)	.758
**Diaphragm thickness insp. left**	Path. (<2mm) [n/%]	3/40 (8%)	1/22 (5%)	1.000	8.107 (0.088-744.034)	.364
**Diaphragm thickening ratio right**	Path. (≤1.5) [n/%]	36/40 (90%)	18/23 (78%)	.267	1.898 (0.125-28.771)	.644
**Diaphragm thickening ratio left**	Path. (≤1.5) [n/%]	36/40 (90%)	17/22 (77%)	.259	16.488 (0.844-322.181)	.065
**Diaphragm thickening fraction right**	Path. (<20%) [n/%]	25/40 (63%)	6/23 (26%)	**.008**	3.986 (0.579-27.447)	.160
**Diaphragm thickening fraction left**	Path. (<20%) [n/%]	19/40 (48%)	5/22 (23%)	.064	3.352 (0.453-24.810)	.236
**Diaphragm excursion right**	Path. (<10mm) [n/%]	25/40 (63%)	7/23 (30%)	**.019**	4.481 (0.659-30.449)	.125
**Diaphragm excursion left**	Path. (<10mm) [n/%]	19/39 (54%)	7/22 (32%)	.282	2.656 (0.399-17.681)	.312

Depicted are number of patients with normal and pathological findings in ultrasound diaphragm measures in regard to normative values according to [1–5]. § *p* values are from Fisher's exact test. # Binary logistic regression analyses were performed to adjust for the candidate covariates sex, age and BMI. The reported regression coefficients (ExpB with 95% confidence interval) >0 indicates the magnitude of influence of disease entity on diaphragm parameters after adjusting for candidate covariates sex, age and BMI and standardization. ALS, Amyotrophic lateral sclerosis; SMA, spinal muscular atrophy; BMI, Body mass index.

References

1. Cardenas, L.Z.; Santana, P.V.; Caruso, P.; Ribeiro de Carvalho, C.R.; Pereira de Albuquerque, A.L. Diaphragmatic Ultrasound Correlates with Inspiratory Muscle Strength and Pulmonary Function in Healthy Subjects. *Ultrasound Med Biol* **2018**, *44*, 786-793, doi:10.1016/j.ultrasmedbio.2017.11.020.
2. Summerhill, E.M.; El-Sameed, Y.A.; Glidden, T.J.; McCool, F.D. Monitoring recovery from diaphragm paralysis with ultrasound. *Chest* **2008**, *133*, 737-743, doi:10.1378/chest.07-2200.
3. Kabitz, H.J.; Walterspacher, S.; Mellies, U.; Criee, C.P.; Windisch, W. [Recommendations for respiratory muscle testing]. *Pneumologie* **2014**, *68*, 307-314, doi:10.1055/s-0034-1365283.
4. Spiesshoefer, J.; Herkenrath, S.; Henke, C.; Langenbruch, L.; Schneppe, M.; Randerath, W.; Young, P.; Brix, T.; Boentert, M. Evaluation of Respiratory Muscle Strength and Diaphragm Ultrasound: Normative Values, Theoretical Considerations, and Practical Recommendations. *Respiration* **2020**, *99*, 369-381, doi:10.1159/000506016.
5. Windisch, W.; Schonhofer, B.; Magnet, F.S.; Stoelben, E.; Kabitz, H.J. [Diagnosis and Treatment of Diaphragmatic Dysfunction]. *Pneumologie* **2016**, *70*, 454-461, doi:10.1055/s-0042-106694.
