# Supplementary figures and images for: Affection of Respiratory Muscles in ALS and SMA
Source: J Clin Med. 2022 Feb 22;11(5):1163. doi: 10.3390/jcm11051163 (PMC8910994; doi:10.3390/jcm11051163)

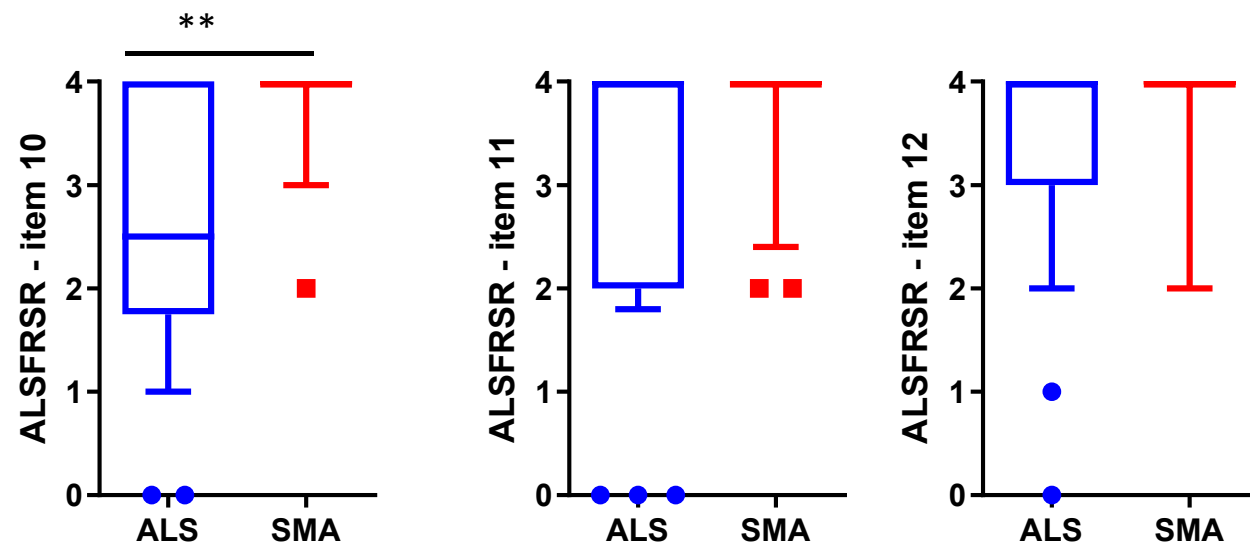

**Figure S1.** Individual items of ALSFRSR respiratory subscores are shown. \*\*  $p$  values < 0.01.

Supplement: Supplementary file 1 [file jcm-11-01163-s001.zip › Figure S1.pdf]
